# Current trends in household food insecurity, dietary diversity, and stunting among children under five in Asia: a systematic review

**DOI:** 10.7189/jogh.15.04049

**Published:** 2025-01-17

**Authors:** Binish Islam, Tasiu Ibrahim Ibrahim, Tingting Wang, Mingyang Wu, Jiabi Qin

**Affiliations:** 1 Xiangya School of Public Health, Central South University, Changsha, Hunan Province, China; 2 Department of Neurological Surgery, The Second Xiangya Hospital of Central South University, Changsha, Hunan Province, China; 3 School of Public Health, Kunming Medical University, Kunming, Yunnan, China

## Abstract

**Background:**

Household food insecurity (HFI) and poor dietary diversity (DD) are major public health challenges in Asia, greatly contributing to stunting among children under five. While previous research has focussed primarily on African regions, this systematic review provides novel insights into the association between HFI, DD, and stunting within the Asian context.

**Methods:**

We searched across six major databases for studies published between 2019 and 2023 exploring the association between HFI, DD, and stunting in children under five across Asia. We then extracted their characteristics, evaluation methods, and outcomes related to stunting for analysis.

**Results:**

From 3215 records, 37 met the inclusion criteria. Most studies were from South Asia (n = 22), followed by Southeast Asia (n = 8), with fewer from West (n = 4), East (n = 2), and Central Asia (n = 1), highlighting geographical research gaps. We found high stunting rates among children under five, especially in South and Southeast Asia. Stunting was strongly linked to HFI and poor DD, suggesting that these factors are critical in addressing malnutrition. Socioeconomic factors, maternal education, and access to clean water also influence stunting outcomes.

**Conclusions:**

Current research on HFI, DD, and stunting in Asia shows substantial variation, with the highest stunting rates in South and Southeast Asia. Limited data from Central and East Asia highlights the need for more comprehensive research in these regions. Addressing HFI and improving DD is critical for reducing stunting and achieving global nutrition targets by 2030.

Optimal nutrition is a fundamental right of every child and is essential for promoting physical growth, development, and overall well-being [1]. Despite global efforts, stunting remains a pressing issue, with 22.3% of children under five years affected worldwide. Asia accounts for 52% of the global burden of stunting and over 75% of children are classified as severely wasted [2,3]. In particular, countries in South and Southeast Asia are burdened with high rates of childhood malnutrition, with stunting and wasting affecting millions of children [4]. Indonesia, the Philippines, and Timor-Leste have the highest stunting rates in Southeast Asia, with prevalence rates of 72.8%, 31%, and 28.8%, respectively [5]. This persistent challenge has been exacerbated by household food insecurity (HFI), which is strongly influenced by poverty, rapid population growth, and environmental factors including climate change [6]. Despite significant efforts to address malnutrition, such as the United Nations Decade of Nutrition (2016–25) and Sustainable Development Goal (SDG) 2, progress remains uneven, particularly in developing regions with stark disparities in access to nutritious food [7].

The factors that determine HFI and dietary diversity (DD) are multifaceted. Inflation, economic instability, and declining agricultural productivity expose millions of households to food insecurity, undermining children's nutritional well-being and increasing the risk of malnutrition [8,9]. Stunting, a key indicator of chronic malnutrition, is similarly influenced by a range of economic, environmental, and socio-cultural factors [10]. For instance, large household sizes, inadequate health services, poor water, sanitation, and hygiene (WASH) practices, and low parental education levels all contribute to poor child nutrition [11,12]. Despite variations in socioeconomic and cultural factors and healthcare systems across Asian countries, a comprehensive comparison of these factors remains crucial to understanding the shared and divergent challenges related to food insecurity and malnutrition across the region [13,14].

The first 24–59 months of life are critical for children's physical and cognitive development [15]. Insufficient nutrition during this period can have irreversible consequences, affecting not only immediate health outcomes, but also long-term academic performance and economic potential [16]. Recent global crises, such as the coronavirus disease 2019 (COVID-19) pandemic, have further highlighted vulnerabilities in food systems, disproportionately affecting marginalised communities and exacerbating existing disparities in child nutrition [17]. These challenges underscore the need for targeted interventions that are sensitive to the unique socioeconomic and cultural contexts across different regions of Asia.

Stunting during early childhood continues to be a significant public health challenge in South and Southeast Asia, where a substantial proportion of children under five remain affected [18–22]. Beyond undermining children's immediate health, the condition has long-term consequences for cognitive development, future productivity, and society [23,24]. Addressing HFI and improving DD are central to addressing stunting and other forms of undernutrition in the region [25], which would subsequently help governments and international organisations can make substantial progress toward Sustainable Development Goal (SDG) 2, which targets the elimination of hunger by 2030 [26].

Despite the importance of understanding the relationships between food insecurity, DD, and stunting, previous research has primarily examined these factors in isolation, often within narrow geographic contexts [9,27–29]. Many studies have focussed on specific continents or sub-regions, leaving a significant gap in our understanding of the broader cross-country context of these interrelationships in Asia [30–33]. Through this study, we seek to address this critical gap by providing a comprehensive multi-country analysis that synthesises evidence from diverse Asian countries. Specifically, we want to explore how variations in HFI and DD across regions contribute to stunting while considering the complex interplay of socioeconomic, environmental, and healthcare factors. Research in South Asia [34,35] and Southeast Asia [36] has shown that dietary patterns and food security levels are deeply shaped by regional, cultural, economic, and environmental contexts, highlighting significant regional variations in the prevalence of malnutrition [5,22,37].

Unlike previous studies that treat food insecurity and DD as isolated factors, this review recognises the multifaceted nature of malnutrition and examines how these factors interact within the broader socioeconomic and cultural dynamics of the region [38–42]. In it, we examine how economic, environmental, and sociocultural dynamics collectively exacerbate stunting, a perspective that remains underexplored in current literature. By adopting a multi-country approach, we provide a more nuanced understanding of the drivers of stunting and elucidate regional and contextual variations that have often been overlooked. This perspective remains underexplored in the literature, which tends to focus on single-country analyses or isolated factors contributing to child malnutrition.

Through this systematic review, we aimed to consolidate existing evidence to better understand the underlying drivers of malnutrition in Asia and propose actionable interventions to reduce its prevalence. Our findings could offer valuable insights for policymakers, researchers, and practitioners in formulating effective strategies to combat malnutrition, improve child health and development across the region, and advance global nutrition goals.

## METHODS

Through this systematic review, we explored the association between HFI, DD, and stunting among children under the age of five in Asia. We adhered to the PRISMA guidelines in reporting our findings [43].

### Information sources, search strategy, and selection criteria

We searched six databases – Web of Science, Medline, Science Direct, Ovid, PubMed, and Google Scholar – focussing on research articles published between 2019 and 2023, limited to studies available in English. The search was conducted using the following combination of keywords: [“young children” OR “under-five children” OR “children”] AND [“diet diversity score” OR “dietary diversity” OR “DD”] AND [“food insecurity” OR “food security” OR “HFIAS”] AND [Asia] AND [“wasting” OR “stunting”]. We defined our inclusion and exclusion criteria per the PICOS framework [44] (**Table 1**).

**Table 1:** PICOS framework

Parameters	Inclusion	Exclusion
**Population**	Children below the age of five	Children above the age of five
**Intervention**	No intervention studies were included; only research examining the association between HFI, DD, and stunting was considered.	All other studies were excluded.
**Comparison**	The continent of Asia	Continents other than Asia
** Outcomes**	Studies published from 2019 to 2023 that examined the relationship between HFI, DD, and stunting were considered.	Studies that reported different outcomes or the same outcome before 2019 and after 2023 were excluded.
**Study design**	Cross-sectional study	Other observational study designs (case-control and cohort), experimental designs (randomised controlled trials, quasi-experimental studies), opinion articles, and editorials were excluded.

DD – dietary diversity, HFI – household food insecurity

### Data extraction

We used the EndNote, version 21 (Clarivate, London, UK) to manage the screening process. Following deduplication, two authors (IB and TII) independently screened the titles and abstracts of retrieved records for relevance, followed by their full-text. At the last step, we screened the references of the included records for any relevant studies.

Two authors (IB and TII) then extracted the following data from the included studies: author(s), year, country, number and age of children, number of households, sample size, location, study purpose, methods for evaluating HFI and DD, stunting, and the final results (HFI/stunting, DD/stunting, and stunting prevalence). Here, HFI refers to insufficient access to adequate, healthy, and nutritious food, resulting from scarcity or limited financial resources, which are critical for development and optimal growth. Meanwhile, DD is assessed by the variety of foods consumed by different groups over a specified period; and (iii) Stunting (HAZ < −2 SD), wasting (weight-for-height Z-scores < −2 SD), and underweight (weight-for-age Z-scores<−2 SD) among children under five years of age (Table S1 in the **Online Supplementary Document**).

We categorised the studies by the countries where they were conducted into Asian sub-regions – West Asia, East Asia, Southeast Asia, South Asia, and Central Asia – according to the United Nations (UN) classification [45].

### Quality evaluation

We evaluated the quality of the included studies using the Joanna Briggs Institute (JBI) checklist for Cross-Sectional Analytical Studies (Table S2 in the **Online Supplementary Document**).

## RESULTS

**Figure 1:**
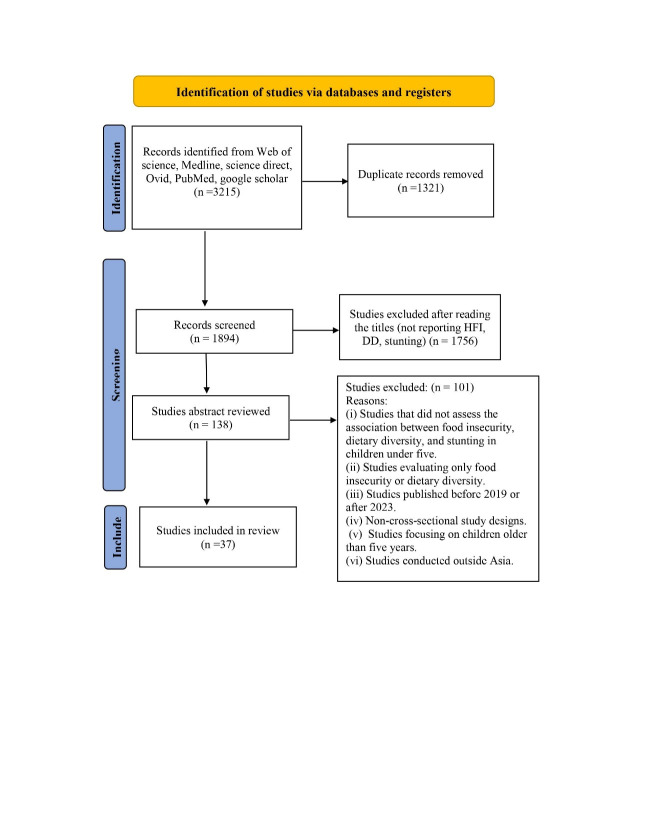
PRISMA flow diagram for the selection process of studies.

We retrieved 3215 records from six different databases, with 1894 remaining after deduplication. We excluded 1756 articles based on titles and abstracts, leaving 138 for full-text screening. Thirty-seven studies met our selection criteria and were included for analysis (**Figure 1**).

### Characteristics of included studies

**Figure 2:**
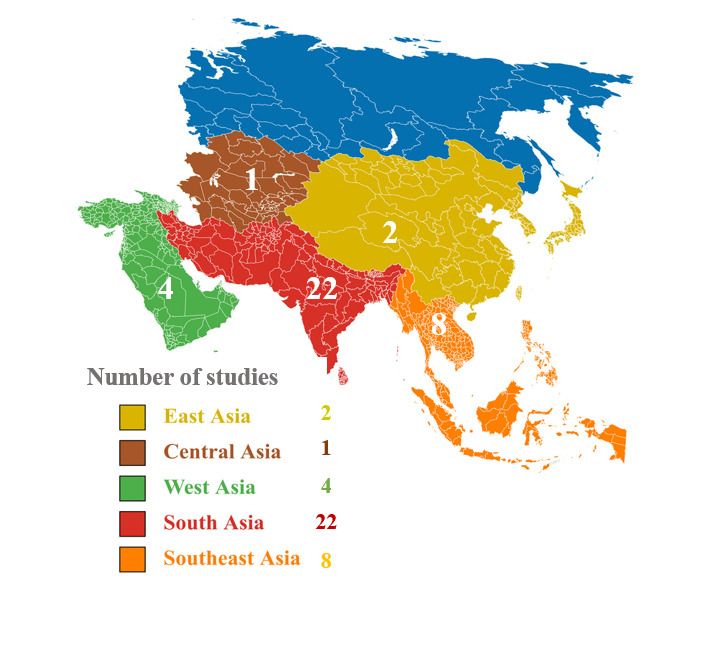
Regional distribution of studies.

Twenty-two studies were carried out in South Asia, eight in Southeast Asia, two in East Asia, one in Central Asia, and four in West Asia (**Figure 2**, Table S1 in the **Online Supplementary Document**). The sample sizes ranged from 120 to 14 216 children, aged between 6 and 59 months; the household samples ranged from 173 to 13 260 households. All studies used standardised methods, such as the Household Food Insecurity Access Scale (HFIAS), and measured stunting using height-for-age Z-scores (HAZ), classifying children as stunted if their HAZ score was below −2 standard deviations (SDs). They otherwise evaluated DD using indicators such as the Minimum Dietary Diversity (MDD), Dietary Diversity Score (DDS), and Dietary Diversity Index (DDI). While DDS quantifies the number of food groups consumed, MDD assesses nutrient adequacy, and DDI evaluates both food group diversity and nutrient quality [46]. Eight studies focussed solely on the association between HFI and stunting, while 19 examined the relationship between DD and stunting. Ten studies assessed HFI and DD concerning stunting.

We observed significant homogeneity among the articles, and we assessed all analyses as being of as high quality using the JBI-MAStARI criteria.

### Evidence from reviewed studies

**Figure 3:**
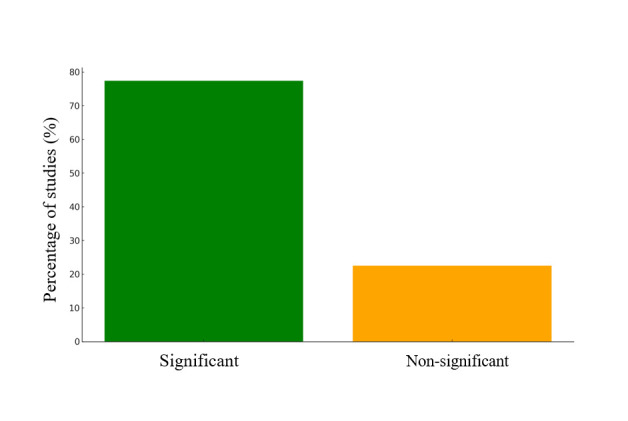
HFI and stunting association.

Among studies examining the association between HFI and stunting, the HFIAS, or other validated measures (**Figure 3****;** Table S1 in the **Online Supplementary Document**), many identified a significant relationship between HFI and stunting [9,27,47–59]. For instance, in Myanmar and Bangladesh, children from food-insecure households had an increased risk of stunting compared to those from food-secure households [54,60]. However, some studies in India and Mongolia reported no significant association between these factors [61,62]. These discrepancies likely arose from variations in food insecurity levels, regional health infrastructure, and methodologies. Regions with stronger public health systems may show less pronounced effects of food insecurity on stunting, thus contributing to the observed inconsistencies. Overall, the data reveal a trend in which stunting rates increase as HFI rises, with notable disparities across regions, particularly in low-income households.

**Figure 4:**
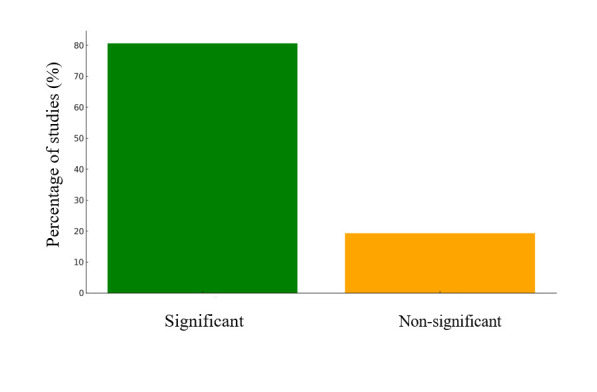
DDS and stunting association.

We observed a strong link between low DD and stunting in most studies (**Figure 4****;** Table S1 in the **Online Supplementary Document**). Studies conducted in Indonesia, Bangladesh, and India showed that children consuming fewer than four food groups had significantly higher odds of stunting [9,27,47,51–53,60,61,63–70]. However, several Southeast and South Asian studies reported no significant association between DD and stunting [48,71–74]. The variability in the findings may stem from differences in dietary assessment methods and regional disparities in food access. In some areas, cultural dietary patterns or limited food variety may obscure the link between DD and stunting. Despite these variations, the results consistently indicate a strong association between higher DD and a reduced prevalence of stunting.

**Figure 5:**
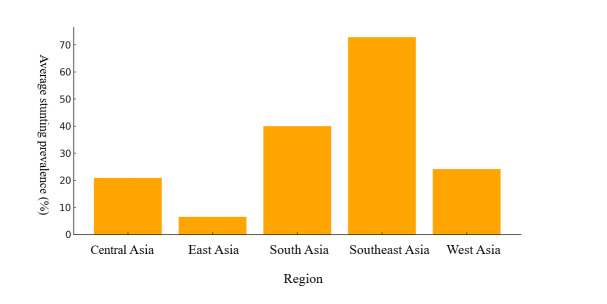
Regional variations in stunting prevalence across Asia.

Stunting rates in the included studies varied widely, from as low as 7.5% in Kuwait [66] to as high as 72.8% in Lao PDR [47]. Southeast Asia had the highest prevalence, reaching approximately 70%, indicating a significant public health concern in that region (**Figure 5**). South Asia followed with around 40%, while Central, East, and West Asia had notably lower rates, ranging from 10% to 20%. Stunting rates were consistently higher in children living in food-insecure households and those with poor DD. In Bangladesh, children with low DD had a significantly higher risk of stunting [53]. These regional disparities in stunting prevalence can be attributed to differences in socioeconomic conditions, access to health care, and regional nutritional programmes. The higher prevalence in Southeast Asia is particularly influenced by ongoing food security challenges and insufficient health care access.

Across Asia, the highest rates of stunting were observed in children under 24 months, suggesting that early childhood is critical for addressing food insecurity and DD.

**Figure 6:**
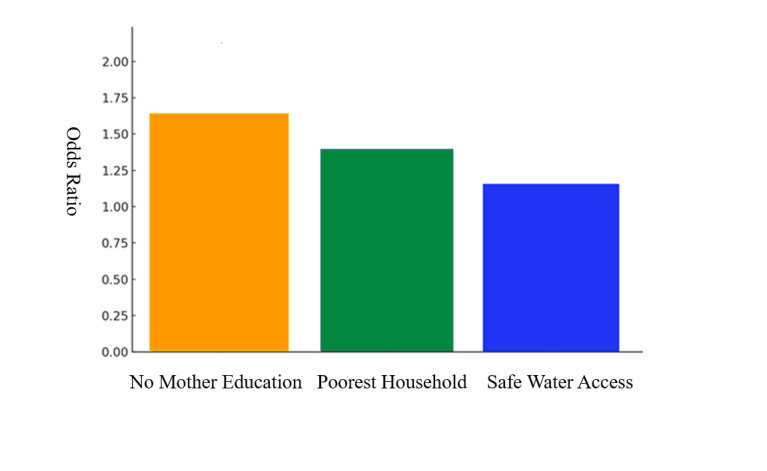
Impact of sociodemographic factors on stunting odds [75].

Sociodemographic factors such as socioeconomic status, mother's education, and the availability of safe water were often cited as contributors to stunting (**Figure 6**). Maternal education was frequently associated with stunting, with lower maternal education levels being linked to higher stunting rates, particularly in food-insecure households. Educated mothers were more likely to manage food resources effectively and ensure better DD for their children, even in food-insecure settings. Similarly, higher household income improved access to a variety of nutritious foods, mitigating the negative impact of food insecurity. These factors interact in complex ways, influencing both food access and the ability to make healthier dietary choices, which in turn affects child growth outcomes. For instance, in Bangladesh and Tajikistan, children of mothers with less education had higher rates of stunting, particularly in food-insecure households [54,65]. Additionally, access to clean water was also a significant factor, with children from households lacking year-round access facing a higher risk of stunting [51].

## DISCUSSION

In this systematic review of 37 studies, we investigated the relationship between HFI and DD with stunting among children under five across Asia. Eight studies in our sample focussed on the relationship between HFI and stunting, 19 examined DD and stunting, while 10 assessed both factors concerning stunting. The key findings from this review are that HFI and poor DD are strongly associated with stunting in children under five across multiple countries in Asia; stunting is the most prominent form of malnutrition associated with food insecurity and poor DD; and that DD may act as a mediating factor between HFI and stunting, particularly in countries with severe food insecurity. Similar findings have been reported in other regions, highlighting the universal nature of this issue. For instance, studies from Colombia, Brazil, and Canada also reported a significant relationship between food insecurity and stunting [76–78], while those in China particularly noted the vulnerability of left-behind children due to restricted access to adequate food resources [9]. This association was significant in the context of South Africa, with food insecurity and limited access to nutritious food acting as major contributors to stunting among children in low-income households [79]. In California, HFI showed a statistically significant impact, particularly in families where the household head had a lower level of education [80]. However, some studies from Asia, such as those conducted in Mongolia and India, reported no significant relationship between HFI and stunting [61,62]. Despite these differences, the overall trend shows that the prevalence of stunted children increases with higher levels of HFI, particularly in low-income households. This highlights the crucial role of both food security and household wealth in influencing stunting rates.

Almost all of the research included in this review reported a significant association between DD and stunting. Our findings align with research conducted in sub-Saharan Africa, where DD has been shown to impact child growth and nutrition significantly [81]. Research in Ethiopia showed that children with poor DD had a higher likelihood of experiencing stunted growth [82]. Similarly, studies from Latin America, particularly in Guatemala, also identified DD as a critical determinant of paediatric growth and development [83]. However, these results contrast with findings from Peru and Tanzania, where some studies reported no significant relationship between DD and stunting in children under five [84,85]. These global comparisons underscore the role of DD and highlight regional variations in its impact on child growth outcomes.

We further found that HFI and DD were strongly correlated with stunting in two-thirds of the selected studies, suggesting these factors are important predictors in this sense. These findings highlight the need to improve household food security and enhance DD to uphold children's nutritional status. However, other critical determinants of stunting, such as access to healthcare, environmental conditions, and cultural practices must also be considered. The prevalence of stunting is worsened by restricted healthcare access, with more than 60% of children in poor countries lacking crucial nutritional support [86,87]. Environmental factors, such as poor sanitation and unsafe water, contribute to 50% of child deaths caused by diarrhoea, which significantly increases the risk of stunting [88]. Cultural practices, such as food taboos, restrict access to diverse and nutritious foods, and 35% of households in some regions report dietary restrictions that negatively impact child nutrition [89,90]. These considerations underscore the need for a comprehensive multidimensional approach to combat stunting, extending beyond food insecurity and DD.

Parental education also emerged as a key factor in our review, whereby lower levels of maternal education were significantly linked to higher rates of stunting. Maternal education affects child nutrition by influencing a mother’s ability to manage HFI and enhance DD [91]. Educated mothers are more likely to possess the knowledge and skills like the infant- and young child-feeding practices necessary for improving food selection, ensuring better nutrient-rich diets, and managing food resources effectively, as found in studies conducted in Myanmar [92], Thailand [93], and even in non-Asian countries like Ghana [94] and Zimbabwe [95]. This increased awareness helps mitigate the negative impacts of food insecurity and poor DD on child growth, particularly in resource-limited settings [96].

This finding also aligns with global studies, including those from Latin America and sub-Saharan Africa, which reported that children whose mothers have low levels of education are at a higher risk of being stunted [97,98]. In Latin America, for example, maternal education was found to be correlated with better child nutrition outcomes, as educated mothers tend to have greater access to nutritional knowledge and health care services [99]. Similarly, higher maternal education in Africa often correlates with improved household income and food security, further protecting against stunting [100].

We also found gender differences in stunting, showing that boys are more commonly affected by stunting than girls. This disparity can be attributed to several factors. In some cultures, especially in northeast India, many communities are matriarchal, meaning that the status of women is usually better [101]. Biologically, boys tend to have higher energy requirements during early childhood, which may increase their susceptibility to stunting due to inadequate nutrition [102]. Moreover, in some regions, social norms may prioritise girls’ nutritional needs, further exacerbating boys’ vulnerability to stunting [103,104].

This trend extends beyond Asia, with similar findings reported in Africa, where boys are often at greater risk of stunting due to cultural and social norms that prioritise the care of girls [105]. Studies conducted in Europe have shown mixed results regarding gender disparities in stunting, with some countries reporting no notable difference between boys and girls [106,107]. These varied findings highlight the necessity for more region-specific research to investigate the gender-related factors influencing stunting across different cultural contexts.

Finally, we found no significant association between marital status and stunting. This aligns with studies from Papua New Guinea that also found no association between marital status and stunting [108]. However, this contrasts with findings from other regions. For instance, research in Norway found a stronger association between stunting and children born to unmarried or young mothers [109]. A study conducted in sub-Saharan Africa similarly observed that children of unmarried mothers faced a higher risk of stunting, likely due to lower household income and limited access to support networks [110,111], while a study conducted in Ethiopia reached the same conclusion [112]. Studies from Italy have shown that maternal age is a key determinant of stunting, with younger maternal age (below 18 years) linked to an increased risk of child malnutrition and negative pregnancy outcomes [113]. These global comparisons suggest that while marital status may not be a strong determinant of stunting in Asia, it could still influence child health outcomes in other contexts.

Urban-rural disparities significantly influence food insecurity, DD, and stunting [114]. For example, in Kuwait (an urban area), despite better access to food and health care, 32% of children in food-insecure households are stunted, while 25% of urban populations face challenges such as food deserts, where access to nutritious, varied foods is limited [66]. In contrast, rural areas report higher levels of food insecurity, with 40% of households in South Asia experiencing inadequate access to diverse, nutrient-rich foods, and stunting rates reaching 40% in rural Bangladesh [53,70]. These disparities highlight the need for tailored interventions that address the unique challenges of both urban and rural populations. Furthermore, the COVID-19 pandemic has worsened food insecurity, especially in low-income households [115]. In India, 47% of households faced food insecurity during the pandemic [116], with 60% of urban and 75% of rural households reporting reduced access to nutritious foods [117]. Similarly, 68% of households in Bangladesh shifted to cheaper, less nutritious foods, increasing the risk of stunting [118]. These disruptions have led to rising stunting rates, especially in children under five years of age [56]. Despite the insights gained from this review, significant data gaps remain, particularly in Central and East Asia. Future research should adopt a longitudinal design to better understand the long-term effects of food insecurity and DD on stunting. Mixed-methods approaches, combining quantitative and qualitative data, can provide deeper insights into regional cultural and socio-economic factors. Collaborative research initiatives and data-sharing networks could also help fill these gaps and enhance the robustness of future studies.

### Recommendations and adoption strategies

Our findings highlight the urgent need for policymakers in Asia to address HFI and poor DD as key contributors to undernutrition in children under five. Strategies should focus on improving food security for low-income households [119], enhancing nutrition education for mothers and children, and strengthening healthcare access. Existing nutritional programmes have focussed on enhancing food security through integrated interventions, such as India's Integrated Child Development Services [120], which combine food distribution with nutrition education. The Bangladesh Nutrition Project has also shown positive results by enhancing DD in rural areas. Further aligning efforts with SDG 2 could also help reduce stunting and promote long-term child health [119].

### Strengths and limitations

This review is among the first to explore the relationship between HFI, DD, and stunting in children under five across Asia. A key strength lies in the comprehensive search strategy applied across multiple databases and the rigorous methodology used to assess studies. However, it also has several limitations, as it only included cross-sectional studies published in English. We also decided to focus exclusively on cross-sectional studies to ensure homogeneity in the study design, allowing for more consistent comparisons. However, this hindered us from estimating causal relationships, which ultimately affected the robustness of our findings and limited our ability to offer policy recommendations. Longitudinal studies would provide stronger evidence of causal relationships and offer more reliable guidance for interventions. Future research using such designs could further substantiate these findings and enhance their applicability to policy.

Our sample also included a small number of studies from certain Asian regions, such as East Asia and Central Asia, which further restricted the generalisability of our findings. The underrepresentation of studies from these regions may be due to factors such as limited research funding, differing health priorities, and insufficient data collection. Addressing this gap requires strengthening research capacity and fostering regional collaborations. Lastly, differences in measurement approaches between studies may have introduced variability, affecting the comparability of findings across studies, thereby influencing the observed strength of the relationship between DD and stunting. Further investigation is necessary to examine the influence of HFI and DD on younger children aged 6–23 months and the effect of gender differences.

## CONCLUSIONS

Our review highlights a significant association between HFI, poor DD, and stunting among children under five in Asia. Inadequate access to nutrient-dense foods and reliance on less diverse diets are central issues contributing to undernutrition in the region. Ineffective implementation of national nutrition programmes and food-sharing practices further exacerbates the problem. Policymakers should adopt comprehensive strategies to improve children's nutritional well-being, including food security measures, better programme delivery, and social behaviour change communication to foster proper feeding practices and overall child nutrition in Asia.

## Additional material


Online Supplementary Document
