# Supplementary material for: Current trends in household food insecurity, dietary diversity, and stunting among children under five in Asia: a systematic review
Source: J Glob Health. 2025 Jan 17;15:04049. doi: 10.7189/jogh.15.04049 (PMC11737815; doi:10.7189/jogh.15.04049)
Supplement: Online Supplementary Document [file jogh-15-04049-s001.pdf]

Supplement to: Islam B, Tasiu II, Wang T, Wu M, Qin J. Current trends in household food insecurity, dietary diversity, and stunting among children under five in Asia: a systematic review. J Glob Health. 2025;15.04049.

Table S1. Overview of selected articles

Author [Ref.], Year, Country	No. of Children (*N0*), Age, No. of Households (*N1*)	Sample/Location	Study Purpose	Evaluation Methods for HFI, DD, and Stunting	HFI and Stunting	DDS and Stunting
			**East Asia**			
Yang et al.[9] 2019 China	N0 = 553 3-5 years N1 = 553	Hunan Province	Examine food insecurity and stunting in left-behind children (LBC).	HFIAS for food insecurity, DDS via 24-hour recall, and FFQ, stunting measured by height-for-age Z-score (HAZ).	Severe HFI is linked to higher stunting (OR = 6.50).	Low DDS is significantly associated with stunting ($p < 0.05$).
Janmohamed et al.[46] 2020 Mongolia	N0 = 938 6–23 months N1 = 938	Nationwide, Mongolia	Assess complementary feeding, dietary diversity, and stunting.	Stunting is measured by HAZ, and DDS per WHO IYCF guidelines.	No significant association.	Higher DDS is associated with reduced stunting risk.
			**Southeast Asia**			
Boulom et al.[47] 2020 Lao PDR	N0 = 173 12-47 months N1 = 173	Nong District, Savannakhet Province	Identify malnutrition and related factors.	FIES for food insecurity, HDDS for dietary diversity, and	90% of households experienced food insecurity; stunting prevalence was 72.8%.	Low DDS is linked to higher stunting rates.

				HAZ for stunting.		
Nai et al. [48] 2020 Indonesia	N0 = 135 6–23 months N1 = 135	Mergangsan Public Health Center, Yogyakarta	Analyze dietary diversity and stunting association.	DDS based on ≥4 food groups, stunting by length-for-age Z-scores.		Poor DDS (<4 food gross-ups) is associated with higher stunting risk (RP: 2.87, CI: 1.23–6.68).
Soe et al.[49] 2023 Myanmar	N0 = 1,207 6–59 months N1 =1207	16 townships spread across 3 regions and 5 states in Myanmar.	Assess dietary diversity and stunting association.	HAZ for stunting, DDS by food groups consumed.		Low DDS (<4 food groups) raised the stunting risk by 1.47x (p = 0.02).
Benedict et al.[50] 2020 Thailand	N0 = 12,313 0-59 months N1 = 12,313	Nationwide, Thailand	Investigate stunting-overweight prevalence and IYCF practices.	Stunting was measured by height-for-age Z-scores; dietary diversity was assessed using the WHO IYCF guidelines.		Dietary diversity has no significant association with stunting.
Mya et al.[51] 2019 Myanmar	N0 = 1,222 6–23 months N1 = 13,260 households	Myanmar	Assess dietary diversity and their association with stunting.	Stunting measured by height-for-age z-scores; minimum dietary diversity		Children who achieved minimum dietary diversity were not

				evaluated based on WHO IYCF guidelines.		significantly less likely to be stunted (p = 0.226).
Martha Irene Kartasurya et al.[52] 2023 Indonesia	N0 = 3,238<br>6-23 months<br>N1 = 3,238	Central Java, Indonesia	Investigate determinants of LAZ scores in children.	Dietary Diversity (DDS): Evaluated through a 24-hour food recall. Stunting: Assessed using Length-for-Age Z (LAZ) scores.		No significant correlation between DDS and stunting.
Omas Bulan Samosir et al.[53] 2023 Indonesia	N0 = 14,216<br>6-23 months<br>N1 =14,216	Indonesia	Assess food diversity and stunting association.	DDS via 8 food groups from RISKESDAS 2018, stunting by WHO standards.		Lower DDS is linked to higher stunting odds.
Basri et al.[54] 2021 Indonesia	N0 = 340<br>2-5 years<br>N1 = 340	Jeneponto District, Indonesia	Assess the relationship between dietary diversity, dietary patterns, and dietary intake and stunting in children.	FFQ for dietary diversity, 24-hour recall method for dietary intake; WHO Anthro for stunting evaluation.	A significant association between food insecurity and stunting.	A significant association between dietary diversity and stunting ($p < 0.05$).
			**Central Asia**			
Barth-Jaeggi et al.[55] 2020	N0 = 2,149<br>6–59 months<br>N1 = 2,160	Tajikistan	Evaluate nutritional status in children and	HAZ for stunting, 24-		DDS contributed to stunting;

Tajikistan			women, focusing on malnutrition.	hour recall for DDS.		20.9% of children were stunted.
			**West Asia**			
Al-Taiar et al.[56] 2020 Kuwait	N0 = 5839 0–23 months N1 = 5839	Kuwait (Vaccination centers)	Investigate dietary diversity and stunting association.	DDS via 24-hour recall, HAZ for stunting by WHO standards.		Minimum DDS is inversely related to stunting (7.5% prevalence in children not meeting criteria).
Abi Khalil et al[57]. 2022 Lebanon	N0 = 384 0–59 months N1 = 384 households	Beirut and Mount Lebanon	Evaluate feeding patterns, dietary diversity, and malnutrition.	DDS via 24-hour recall, HAZ for stunting by WHO standards.		32% of children with low DDS were more likely to be stunted.
El Bilbeisi et al.[27] 2022 Palestine (Gaza Strip)	N0 = 350 <5 years N1 = 350 households	Gaza Strip, Palestine	Assess food insecurity, dietary diversity, and malnutrition in children under five.	HAZ for stunting, Radimer/Cornell scale for food insecurity, 24-hour recall for DDS.	32.8% of food-insecure children were stunted, vs. 18.0% in food-secure households.	95.2% of food-insecure children had low DDS; low DDS was linked to stunting.
Sotoudeh et al.[58] 2021 Iran	N0 = 421 2-6 years N1 = 421	Southeast of Iran	Examine food insecurity and sociodemographic	USDA Food Security Module for HFI, anthropometric	A significant association between HFI and stunting.	

			factors in children.	measurements for stunting.		
			**South Asia**			
Hasan et al.[59] 2023 Bangladesh	N0 = 26,353 6–59 months N1 = 77,036	Nationally representative, FSNSP data	Assess seasonal variations in HFI and child nutrition, with a focus on complementary feeding (CDD).	HFIAS for food insecurity, DDS via the WHO 7-item scale, and HAZ for stunting.	HFI is positively associated with stunting (AOR: 1.12).	
Choudhary et al.[60] 2021 India	N0 = 58038 6-23 months N1 = 58038	National sample, India	DDS measured by 7 food groups, HAZ for stunting.	Household water insecurity is indirectly linked to stunting.	Household water insecurity indirectly contributes to child stunting.	Lower DDS increased stunting likelihood.
Mitra et al.[61] 2019 India	N0 = 190 Under 5 years N1 = 190	240/ Bankura Municipality, West Bengal	Explore household food security, nutrient adequacy, and stunting.	HHFS scale for HFI, 24-hour recall for nutrient adequacy, IDDS-16 for DDS.	Households with lower food security had higher stunting rates.	Lower DDS (≤3 food groups) linked to stunting.
Roshania et al.[62] 2022 India	N0 = 2,564 0-35 months N1 = 2,564	Bihar, India	Assess migration's association with stunting and wasting in children.	HAZ for stunting, FIES for HFI, DDS for dietary evaluation.		Limited DDS linked to stunting; only 4.3% of children consumed ≥4 food groups.
Pandey et al.[63] 2021	N0 = 5,772 6–23 months N1 = 5,772	Nationwide, India	Explore the effects of dairy intake and DDS	HAZ for stunting, DDS via 24-hour		Minimum DDS associated

India			on child anthropometrics.	recall, and WHO IYCF guidelines.		with 1.37x higher stunting risk.
Ali et al.[64] 2019 Bangladesh	N0 = 6,468 6–59 months N1 = 8,679	Rural Bangladesh (multiple districts)	Examine HFI, socio-economic factors, and stunting.	Stunting was measured by height-for-age z-scores, and DDS was calculated from a 24-hour recall of food groups.	Children from food-insecure households had significantly increased odds of stunting.	Children with higher DDS had significantly lower odds of stunting.
Iqbal et al.[65] 2019 Bangladesh	N0 = 324 24–36 months N1 = 324	Urban slum community, Dhaka	Assess DDS and stunting association.	Stunting was assessed using height-for-age z-scores, while dietary intake was evaluated through a semi-quantitative food frequency questionnaire.		Lower dietary diversity was significantly associated with stunting.
Ahmed Jubayer et al.[66] 2022 Bangladesh	N0 = 256 Under 5 years old N1 = 256	St. Martin’s Island, Bangladesh	Investigate malnutrition prevalence and related factors.	FIES for HFI, WHO Anthro for stunting.	A significant association between food insecurity and stunting.	
Pathak et al.[67] 2020 India	N0 = 510 Preschool children N1 = 510	Dibrugarh district, Assam	Evaluate HFI and nutritional status.	HFIAS for HFI, anthropometric measures for stunting, wasting, and underweight.	No Significant association between HFI and stunting	

Burman et al.[68] 2022 India	N0 = 257 Under 5 years old N1 = 257	Kolkata, West Bengal, India	Investigate HFI and child nutrition.	Household Food Insecurity Access Scale (HFIAS), WHO Anthro for stunting.	A significant association between HFI and stunting.	
Sabu et al.[69] 2020 India	N0 = 314 2-5 years N1 = 314	Wayanad district, Kerala	Examine inequality in undernutrition among Paniya and Kurichiya tribal communities.	HFIAS for HFI, Composite Index of Anthropometric Failure (CIAF) for stunting.	A significant association between HFI and stunting.	
Sajid Bashir Soofi et al.[70] 2023 Pakistan	N0 = 52,602 0-59 months N1 = 115,600	Pakistan	Identify determinants of stunting in children under five	Stunting is measured by Z-score (HAZ) Household food insecurity was measured through a household-level assessment.	40% stunting prevalence in food-insecure households.	
Kim et al.[71] 2019 India	N0 = 140,444 6-59 months N1 = 140,444	National Family Health Survey	Assess 23 correlates of child anthropometric failures (stunting, underweight, and wasting) in India.	Dietary diversity score based on 24-hour recall and stunting measured by WHO growth standards.		Poor dietary diversity associated with stunting.

Houghton et al.[72] 2020 India	N0 = 120<br>12-24 months<br>N1 = 120	Slums of South Delhi, India	Assess feeding, hygiene, and nutrition in disadvantaged children.		Significant association between food insecurity and stunting.	
Satapathy et al.[73] 2021 India	N0 = 360<br>6-23 months<br>N1 = 360	Urban slums of Berhampur, Odisha	Assess malnutrition risk in children.		Significant association between food insecurity and stunting.	A significant association between dietary diversity and stunting ($p < 0.05$).
Chhabra et al. [74]2021 India	N0 = 350<br>6-23 months<br>N1 = 350	East Delhi	To determine the nutritional health of children aged 6–23 months.	HFIAS for HFI, Minimum DDS, WHO Anthro for stunting.	A significant association between food insecurity and stunting.	A significant association between dietary diversity and stunting ($p < 0.05$).
Cunningham et al.[75] 2019 Nepal	N0 = 1,402<br>6–24 months<br>N1 = 4,080	16 districts, in rural Nepal	To identify Association between dietary diversity and stunting.	24-hour dietary recall for DD. Z-scores (LAZ) for stunting.		No significant association between dietary diversity and stunting.
Jeyakumar et al.[76] 2022 India	N0 = 1,443<br>0–24 months<br>N1 = 1,443	Urban slums in Pune, Maharashtra	Examine IYCF practices and malnutrition in slum children.	HFIAS for HFI, 24-hour recall for DDS, WHO Anthro for stunting.		Low DDS is significantly associated with stunting ($p < 0.05$).

Beckerman-Hsu et al.[77] 2020 India	N0 = 67,247<br>6-23 months<br>N1 = 67, 247	Nationwide, India	Assess dietary and anthropometric failures among children.	HFIAS for HFI, 24-hour recall for DDS, WHO Anthro for stunting.		Low Dietary diversity linked to stunting (*p* < 0.05).
Loukrakpam et al.[78] 2020 India	N0 = 701<br>Under 5 years<br>N1 = 1,920	Meitei community, Manipur	Investigate food consumption patterns and nutritional status.	DDS, 24-hour recall, anthropometric measurement for stunting.		Low dietary diversity has a significant association with stunting.
Gupta et al.[79] 2020 India	N0 = 259,627<br>0–5 years<br>N1 = 601,509	India	Examine correlates of childhood stunting.	DDS via NFHS-4 data, WHO standards for stunting.		Low dietary diversity associated with stunting (*p* < 0.05
Ravindranath et al.[80] 2019 India	N0 = 131<br>Under 5 years<br>N1 = 131	Ahmedabad, Gujarat	Assess the nutritional status and causes of poor nutrition.	Anthropometric measurements, observations, in-depth interviews.		Lack of DDS a significant cause of stunting (40.5% prevalence).

HAZ: Height-For-Age, DDS: Dietary Diversity Score, DD: Dietary Diversity, HFI: Household food insecurity, HFIAS: Household Food Insecurity Access Scale.

Table S2. Risk of bias in the included studies assessed by JBI-MAStARI

Author, Year, Ref	Q1	Q2	Q3	Q4	Q5	Q6	Q7	Q8	Yes Score (_/8) Methodological quality
Yang Q. et al. 2019 [10]	Y	Y	Y	Y	Y	Y	N	Y	7/8
Janmohamed A. et al. 2020 [20]	Y	Y	Y	Y	Y	Y	Y	Y	8/8
Boulom S. et al. 2020 [21]	Y	Y	Y	Y	Y	Y	N	Y	7/8
Nai HME. et al. 2020 [22]	Y	Y	Y	Y	Y	Y	N	Y	7/8
Soe TK. et al. 2023 [23]	Y	Y	Y	Y	Y	Y	Y	Y	8/8
Benedict L. et al. 2021 [24]	Y	Y	Y	Y	Y	Y	N	Y	7/8
Mya KS. et al. 2019 [25]	Y	Y	Y	Y	Y	Y	Y	Y	8/8
Kartasurya MI. et al. 2023 [26]	Y	Y	Y	Y	Y	Y	N	Y	7/8
Samosir OB. et al. 2023 [27]	Y	Y	Y	Y	Y	Y	Y	Y	8/8
Basri H. et al. 2021 [28]	Y	Y	Y	Y	Y	Y	N	Y	7/8
Barth-Jaeggi T. et al. 2020 [29]	Y	Y	Y	Y	Y	Y	Y	Y	8/8
Al-Taiar A. et al. 2020 [30]	Y	Y	Y	Y	Y	Y	Y	Y	8/8

Abi Khalil H. et al. 2022 [31]	Y	Y	Y	Y	Y	Y	N	Y	7/8
El Bilbeisi AH. et al. 2022 [32]	Y	Y	Y	Y	Y	Y	Y	Y	8/8
Sotoudeh M. et al. 2021 [33]	Y	Y	Y	Y	Y	Y	Y	Y	8/8
Hasan MM. et al. 2023 [34]	Y	Y	Y	Y	Y	Y	Y	Y	8/8
Choudhary N. et al. 2021 [35]	Y	Y	Y	Y	Y	Y	N	Y	7/8
Mitra S. et al. 2019 [36]	Y	Y	Y	Y	Y	Y	Y	Y	8/8
Roshania RP. et al. 2022 [37]	Y	Y	Y	Y	Y	Y	Y	Y	8/8
Pandey S. et al. 2021 [38]	Y	Y	Y	Y	Y	Y	Y	Y	8/8
Ali NB. et al. 2019 [39]	Y	Y	Y	Y	Y	Y	N	Y	7/8
Iqbal MS. et al. 2019 [40]	Y	Y	Y	Y	Y	Y	Y	Y	8/8
Jubayer A. et al. 2022 [41]	Y	Y	Y	Y	Y	Y	Y	Y	8/8
Pathak J. et al. 2020 [42]	Y	Y	Y	Y	Y	Y	Y	Y	8/8
Burman J. et al. 2022 [43]	Y	Y	Y	Y	Y	Y	N	Y	7/8
Sabu KU. et al. 2020 [44]	Y	Y	Y	Y	Y	Y	Y	Y	8/8

Soofi SB. et al. 2023 [45]	Y	Y	Y	Y	Y	Y	Y	Y	8/8
Kim R. et al. 2019 [46]	Y	Y	Y	Y	Y	Y	Y	Y	8/8
Houghton LA. et al. 2020 [47]	Y	Y	Y	Y	Y	Y	N	Y	7/8
Satapathy DM. et al. 2021 [48]	Y	Y	Y	Y	Y	Y	Y	Y	8/8
Chhabra P. et al. 2021 [49]	Y	Y	Y	Y	Y	Y	Y	Y	8/8
Cunningham K. et al. 2019 [50]	Y	Y	Y	Y	Y	Y	Y	Y	8/8
Jeyakumar A. et al. 2022 [51]	Y	Y	Y	Y	Y	Y	Y	Y	8/8
Beckerman-Hsu JP. et al. 2020 [52]	Y	Y	Y	Y	Y	Y	Y	Y	8/8
Loukrakpam B. et al. 2020 [53]	Y	Y	Y	Y	Y	Y	Y	Y	8/8
Gupta AK. et al. 2020 [54]	Y	Y	Y	Y	Y	Y	Y	Y	8/8
Ravindranath D. et al. 2019 [55]	Y	Y	Y	Y	Y	Y	N	Y	7/8

The items were collapsed into 8 quality-appraisal criteria (Q1-Were the criteria for inclusion in the sample clearly defined? Q2-Were the study subjects and the setting described in detail? Q3-Was the exposure measured validly and reliably? Q4-Were objective standard criteria used for measurement of the condition? Q5Were confounding factors identified? Q6-Were strategies to deal with confounding factors stated? Q7-Were the outcomes measured validly and reliably? Q8-Was appropriate statistical analysis used?).

JBI-MAStARI was used to evaluate the risk of bias. *Articles* with scores between 1 and 2 were defined as poor methodological quality, articles with scores between 3 and 4 as moderate quality, and articles with scores more than 5 as high quality.

N, no; NA = irrelevant; U = unsure; Y = yes.
